# Comparative Genomics of a *Helicobacter pylori* Isolate from a Chinese Yunnan Naxi Ethnic Aborigine Suggests High Genetic Divergence and Phage Insertion

**DOI:** 10.1371/journal.pone.0120659

**Published:** 2015-03-23

**Authors:** Yuanhai You, Lihua He, Maojun Zhang, Jianzhong Zhang

**Affiliations:** 1 State Key Laboratory for Infectious Disease Prevention and Control, National Institute for Communicable Disease Control and Prevention, Chinese Center for Disease Control and Prevention, Beijing, China; 2 Collaborative Innovation Center for Diagnosis and Treatment of Infectious Diseases, Hangzhou, Zhejiang, China; USDA-ARS-ERRC, UNITED STATES

## Abstract

*Helicobacter pylori* is a common pathogen correlated with several severe digestive diseases. It has been reported that isolates associated with different geographic areas, different diseases and different individuals might have variable genomic features. Here, we describe draft genomic sequences of *H*. *pylori* strains YN4-84 and YN1-91 isolated from patients with gastritis from the Naxi and Han populations of Yunnan, China, respectively. The draft sequences were compared to 45 other publically available genomes, and a total of 1059 core genes were identified. Genes involved in restriction modification systems, type four secretion system three (TFS3) and type four secretion system four (TFS4), were identified as highly divergent. Both YN4-84 and YN1-91 harbor intact *cag* pathogenicity island (*cagPAI*) and have EPIYA-A/B/D type at the carboxyl terminal of *cagA*. The *vacA* gene type is s1m2i1. Another major finding was a 32.5-kb prophage integrated in the YN4-84 genome. The prophage shares most of its genes (30/33) with *Helicobacter pylori* prophage KHP30. Moreover, a 1,886 bp transposable sequence (IS605) was found in the prophage. Our results imply that the Naxi ethnic minority isolate YN4-84 and Han isolate YN1-91 belong to the hspEAsia subgroup and have diverse genome structure. The genome has been extensively modified in several regions involved in horizontal DNA transfer. The important roles played by phages in the ecology and microevolution of *H*. *pylori* were further emphasized. The current data will provide valuable information regarding the *H*. *pylori* genome based on historic human migrations and population structure.

## Introduction

*Helicobater pylori* (*H*. *pylori*) is a well-defined pathogen that may be correlated with several digestive diseases such as gastritis, ulcers and gastric cancer [1–3]. It is well known that *Helicobacter pylori* can be divided into seven subpopulations with distinct geographical distributions based on the sequence diversity of seven house-keeping genes. Traces of human migrations could be well reflected and integrated with historical stories or events [4–6] by the gene pools of the different populations. Based on several population-level or individual analyses, it was noted that *H*. *pylori* has a high genetic diversity characterized by horizontal transfer and recombination [7–15]. DNA delivery is affected by several type four secretion systems, such as the *comB* system, TFS3 and TFS4. Cryptic plasmids and prophages were also considered as potential vectors for the transfer of DNA across different strains. However, the mechanisms of these transfers are not well understood [16–21].

Since strain 26695 was first sequenced in 1999, exploration of the comprehensive mechanisms contributing to the flexibility of pathogenesis at the genomic level have been ongoing. Especially during the last three years, there has been a dramatic increase of genomic sequences of *Helicobacter pylori* in the GenBank genomics database (45 complete sequences up to May 2013, when the analysis for this study began). As the rapid development of next generation sequencing technology has generated many genome sequences [22–30], more studies are being focused on the investigation of potential molecular mechanisms. However, only one of these complete sequences originated from China. We have reported draft genome sequences for three *H*. *pylori* strains isolated from Heilongjiang province, which has quite a high incidence of gastric disease [31, 32]. Additionally, we recently completed draft genomes of two other isolates recovered from gastritis patients from Yunnan province. Yunnan, a province located in southwest China, borders Myanmar, Laos, and Vietnam and has many local ethnic minorities. Lijiang is located on the Northwest Yunnan Plateau adjacent to the southeast side of the Tibetan Plateau, which is considered to be the "Roof of the World". One of the largest populations in Yunnan is the Naxi population, numbering approximately 300,000, who mainly inhabit the Lijiang Naxi Autonomous County. The Naxi inhabit a relatively limited area and have a particular lifestyle quite different from that of other ethnic groups. Moreover, reports indicate that this area has a high *H*. *pylori* infection rate. Another large population, the Han, mainly inhabit the eastern areas of Yunnan, Kunming. In this study, we performed a global overview of genomic features for the isolates from Yunnan and other publicly available genomes worldwide. The results provide useful information on the genomic diversity of *H*. *pylori* among different locations and ethnicities.

## Materials and Methods

### *H*. *pylori* strains for genome sequencing

Two isolates from Yunnan province were sequenced in a comprehensive study. YN4–84 was isolated from a Naxi gastritis patient. YN1–91 was isolated from a Han gastritis patient. The strains were cultured on a Columbia agar base supplemented with 5% sheep blood, and DNA was extracted as previously described [31, 32].

### Ethics Statement

All patients involved gave informed consent for the use of the samples in studies in writing, and ethical approval was obtained from the ethics committee of the Chinese Center for Disease Control and Prevention (China CDC) and the academic committee of the National Institute for Communicable Disease Control and Prevention, China CDC.

### Genome sequencing

Whole-genome sequencing was performed for each strain using the Illumina HiSeq 2000 by generating paired-end libraries (500 bp and 2 kb) following the manufacturer’s instructions. The read lengths were 90 bp and 50 bp for each library, from which more than 100 Mb of high-quality data were generated. Next, the paired-end reads from the two libraries were assembled de novo into scaffolds. Gene prediction was performed using Glimmer. The tRNA genes were identified with tRNAScan-SE2. rRNA genes were identified with RNAmmer3. The best result for each BLAST search was imported as the gene annotation.

### Genomic data deposition

This whole-genome shotgun project was deposited at the DDBJ/EMBL/GenBank under the accession numbers JPXD00000000 (YN4–84), and JPXC01000000 (YN1–91).

### Gene annotation and comparative genomics

**Fig 1:**
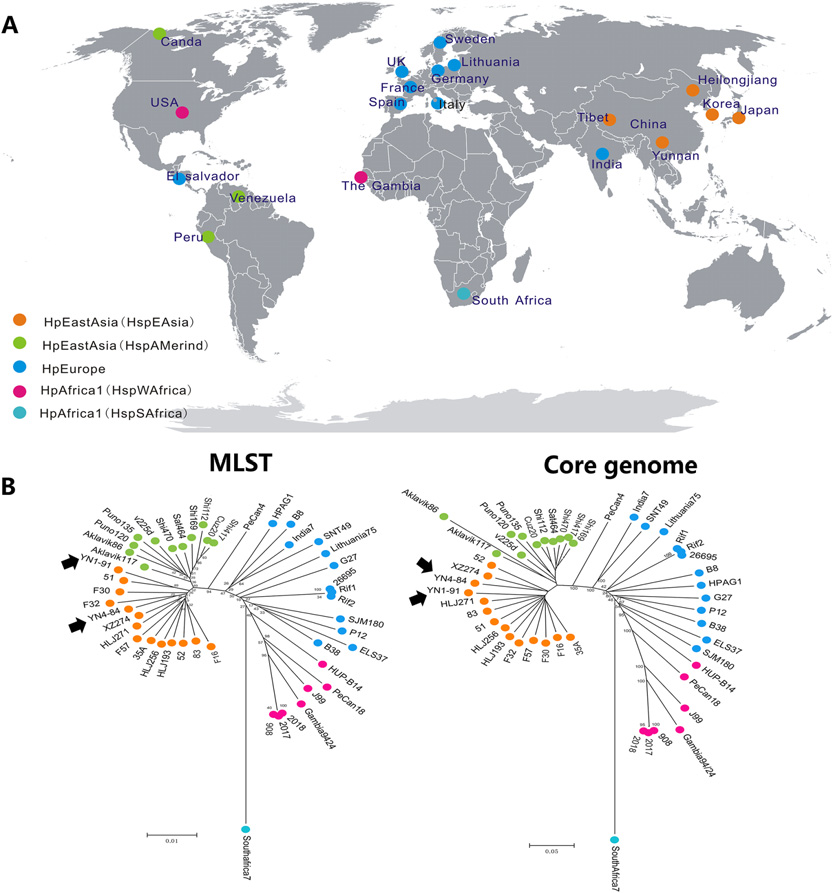
A. Global distribution of sequenced *H*. *pylori* strains from different populations B. phylogenetic comparison based on seven house-keeping genes (Left) and core genomes (Right).

The sequences were uploaded to the RAST server for gene identification and automatic annotation [33]. A total of 47 genome sequences were further analyzed. These sequences were designated on a world map according to their geographic location (Fig. 1A). Detailed background information for these sequenced strains is shown in Table 1. To identify specific regions in YN4–84, YN4–84 was compared with YN1–91 using MAUVE [34]. GVIEW was used to determine the core genomes of the analyzed strains. BLASTATLAS was used to identify variable genome regions based on a comparison of YN4–84 with an additional forty-six genomes [35]. BLAST parameters were set as follows: Expected cutoff value 1e-10, Alignment length cutoff 100bp, Percent identity cutoff 85%. To identify genomic variable regions among all analyzed genomes, P12 was used as a seed genome for pan-genome construction. P12 has a relatively large genome size and dramatic genome diversity. Therefore using P12 as a reference genome might reflect the variation more comprehensively, although it is not the most phylogenetically close strain to YN4–84. The most diverse genomic regions among the 47 sequences were labeled in an arc line. YN4–84 was in the outermost circle. Neighboring YN4–84 were XZ274 and YN1–91.

**Table 1:** Genomic features and backgrounds of the 47 *H*. *pylori* strains used in this study

strain	ACCESSION	length	origin	Clinical diagnosis
26695	AE000511	1667892	UK	Gastritis
J99	AE001439	1643831	USA	Duodenal ulcer
HPAG1	CP000241	1596366	Sweden	Atrophic gastritis
P12	CP001217	1673813	German	Duodenal ulcer
G27	CP001173	1652982	Italy	NA
Shi470	CP001072	1608548	Peru	Gastritis
Shi169	NC_017740	1616909	Peru	NA
Shi417	NC_017739	1665719	Peru	NA
Shi112	NC_017741	1663456	Peru	NA
35A	CP002096	1566655	Japan	NA
51	CP000012	1589954	Korea	Duodenal ulcer
52	CP001680	1568826	Korea	NA
908	CP002184	1549666	France	Duodenal ulcer
B38	FM991728	1576758	France	MALT lymphoma
B8	FN598874	1673997	unknown	Gastric ulcer
Cuz20	CP002076	1635449	Peru	NA
Gambia94/24	CP002332	1709911	The Gambia	NA
India7	CP002331	1675918	India	Peptic ulcer
Lithuania75	CP002334	1624644	Lithuania	NA
PeCan4	CP002074	1629557	Peru	Peruvian gastric cancer
PeCan18	NC_017742	1660685	Peru	Peruvian gastric cancer
SJM180	CP002073	1658051	Peru	Gastritis
Sat464	CP002071	1560342	Peru	NA
SouthAfrica7	CP002336	1501960	South Africa	NA
v225d	CP001582	1588278	Venezuela	Gastritis
2017	CP002571	1548238	France	Duodenal ulcer
2018	CP002572	1562832	France	Duodenal ulcer
F16	AP011940	1575399	Japan	Gastritis
F30	AP011941	1570564	Japan	Duodenal ulcer
F32	AP011943	1578824	Japan	Gastric cancer
F57	AP011945	1609006	Japan	Gastric cancer
83	CP002605	1617426	unknown	NA
XZ274	NC_017926	1634138	China	Gastric cancer
Rif1	CP003905	1667883	German	NA
Rif2	CP003906	1667890	German	NA
Puno120	NC_017378	1624979	Peru	Gastritis
Puno135	NC_017379	1646139	Peru	Gastritis
HUP-B14	NC_017733	1599280	Spain	NA
ELS37	NC_017063	1664587	El Salvador	Gastric cancer
SNT49	NC_017376	1607577	India	Asymptomatic
Aklavik86	CP003476	1494183	Canada	NA
Aklavik117	CP003483	1614447	Canada	NA
HLJ193	ALJI00000000	1552322	China	Atrophic gastritis
HLJ256	ALKA00000000	1576324	China	Atrophic gastritis
HLJ271	ALKB00000000	1588141	China	Gastric ulcer
YN1–91	JPXC01000000	1609835	China	Gastritis
YN4–84	JPXD00000000	1633405	China	Gastritis

### Phage prediction

Possible phage sequences were predicted using PHAST [36]. Information on *H*. *pylori* phages from other strains were imported from the database in PHAST (http://phast.wishartlab.com/Download.html, update: Jan 1 2014). PHAST can predict phage completeness based on a score calculation system. The criteria for scoring prophage regions as intact, questionable, or incomplete was described in detail in the website above. If a region's total score is less than 70, it is designated incomplete; if between 70 to 90, it is designated questionable; if greater than 90, it is designated intact.

### Phylogenetic analysis

Seven house-keeping genes were extracted from these genome sequences and aligned with concatenation to construct a phylogenetic tree using MEGA5 [37]. MUMMER3 was used to perform genome comparison to identify core genome SNPs [38]. Based on a core genome SNP analysis of 47 *H*. *pylori* strains distributed in various worldwide regions, a phylogenetic tree was generated to show the YN4–84 and YN1–91 subtype. Groups of the 47 sequenced strains were identified with different colors based on the phylogenetic analysis of the house-keeping gene sequences and core genome SNPs.

### Virulence gene analysis for YN4–84 and YN1–91

To characterize the virulence of Yunnan isolates, we extracted gene clusters of the *cag* pathogenetic island from the 34 sequenced strains that harbored the intact island and aligned the sequences. We built a neighbor-joining tree based on the sequence diversity of both *cagPAI* and *cagA*. We compared the EPIYA motif among the 34 sequences. We also searched for other virulence genes, including *sabA*, *babA*, *vacA*, *iceA1*, *iceA2*, *oipA*, *dupA* and *homB*.

## Results

### General genomic features of the two Yunnan isolates

**Fig 2:**
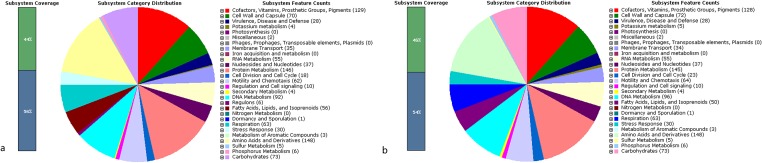
a. Subsystem distribution statistics of *Helicobacter pylori* strain YN4–84 generated by the rapid annotation using a subsystem technology server. b. Subsystem distribution statistics of *Helicobacter pylori* strain YN1–91 generated by the rapid annotation using a subsystem technology server.

We obtained 11 scaffolds with a total length of 1,609,835 bp for the draft genome of strain YN1–91. For strain YN4–84, we obtained 9 scaffolds with a total length of 1,633,405 bp. The average genomic GC content for each strain was 38.5%. The subsystem distribution and general information about the potential functional distribution of YN4–84 and YN1–91are shown in Fig. 2.

### Comparative genomic and phylogenetic analysis

We extracted seven house-keeping genes from these genome sequences and concatenated them to a length of 3,404 bp and used them to construct a neighbor-joining tree. We found a total of 8,644 core SNPs among the 47 analyzed genome sequences. Based on the phylogenetic analysis of the house-keeping gene sequences and core genome SNPs, the groups of the 47 sequenced strains are identified with different colors in Fig. 1B (left) and Fig. 1B (right).

**Fig 3:**
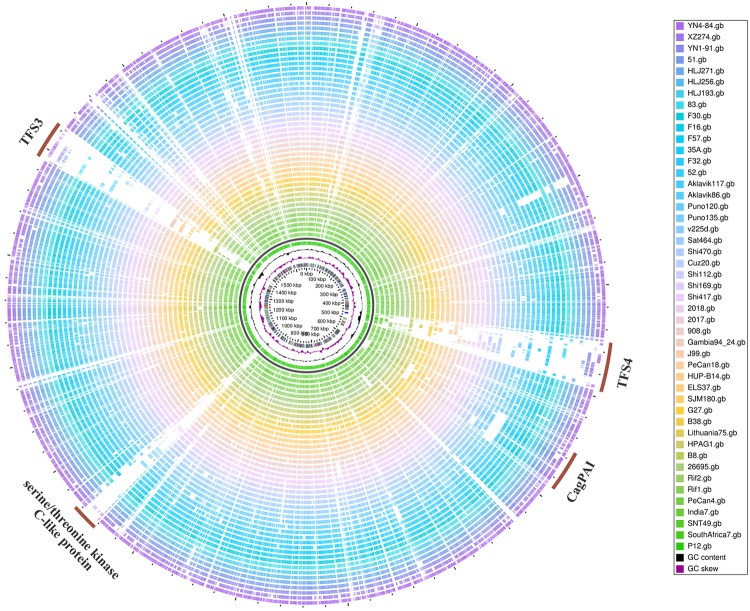
Global overview of genomic diversity of YN4–84 and YN1–91 with other 46 *H*. *pylori* genome sequences. The four regions with most divergence were labeled with brown arcs along the chromosome.

Fig. 3 shows a global overview of genomic diversity of YN4–84 with other 46 *H*. *pylori* genome sequences. The regions showing high diversity were quite consistent with our previous study, from which twelve variable genomic regions were identified based on a high density genome tiling microarray technology. In a picture from BLASTATLAS, four regions with the greatest genome diversity were labeled with a brown arc line. The gene clusters from these regions mainly encoded the type IV secretion system 4, the type IV secretion system 3, the cag pathogenicity island and a serine/threonine kinase C-like protein. Genes coding for restriction modification system enzymes were also quite different in these genomes. Both YN4–84 and YN1–91 have an intact *cag* pathogenetic island. YN4–84 has an intact TFS3 and partial TFS4 system, while YN1–91 has incomplete TFS3 and TFS4 systems. For the core genome analysis, we found 1059 core genes in total present in all of the sequenced strains.

### Phage sequence analysis

**Fig 4:**
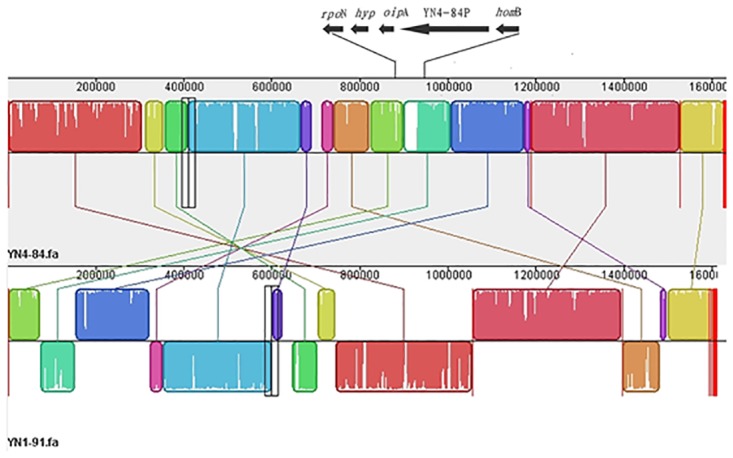
Global alignment of YN4–84 and YN1–91. The black arrows above YN4–84 show the insertion of YN4–84P and its flanking gene fragments.

**Fig 5:**
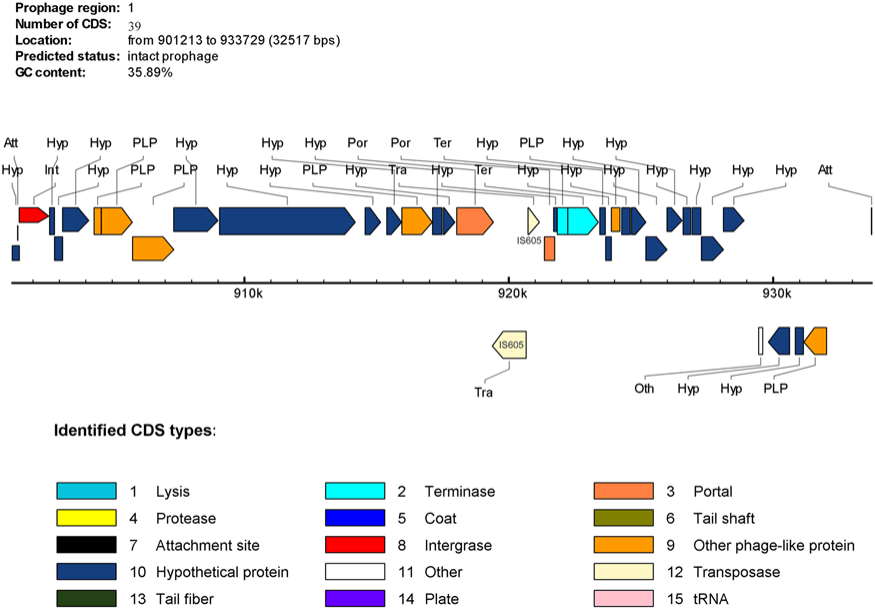
Gene content of YN4–84P.

By comparing YN4–84 with the isolate recovered from the Han patient (YN1–91), we found a large insertion fragment 32,517 bp in length in YN4–84 (Fig. 4). This fragment was also determined as a strain specific region for YN4–84 compared to the other 45 genome sequences available in GenBank. Further analysis of this fragment in Genbank showed it had a high sequence homology with a reported *H*. *pylori* phage (KHP30). This was also confirmed preliminarily by the results of a PHAST analysis. PHAST indicated that the strain specific region was 32,517 bp in length and had 39 predicted CDSs (Table 2). We designated this new phage as YN4–84P. According to the criteria for scoring prophage regions [36], YN4–84P was identified as an intact phage with a high score of 140. The G+C percentage was 35.62%, which was lower than that for the YN4–84 genome (38.43%). The left flanking region was a gene encoding RNA polymerase sigma-54 factor, a transcription factor required for the expression of several flagellar genes. The right flanking region was a homB gene that is reported to have a high positive ratio in gastric cancer isolates. Most of the predicted genes (30/33) were homologous with genes from KHP30. Unlike KHP30, YN4–84P had a 1886 bp transposable sequence (IS605) inserted into the prophage sequence (Fig. 5).

**Table 2:** Gene contents of the predicted phage YN4–84P.

code	CDS_POSITION	BLAST_HIT	E-VALUE
1	901213..901485	PHAGE_Helico_KHP30_NC_019928: hypothetical protein; PP_00960; phage(gi431810541)	1.00E-25
2	901416..901427	attL TCAAAAAACCAC	0
3	901478..902599	PHAGE_Helico_KHP30_NC_019928: putative phage integrase; PP_00961; phage(gi431810542)	7.00E-148
4	902626..902811	PHAGE_Helico_KHP30_NC_019928: hypothetical protein; PP_00962; phage(gi431810543)	3.00E-22
5	902813..903121	PHAGE_Helico_KHP30_NC_019928: hypothetical protein; PP_00963; phage(gi431810544)	1.00E-46
6	903118..904113	PHAGE_Helico_KHP30_NC_019928: hypothetical protein; PP_00964; phage(gi431810545)	2.00E-167
7	904311..904577	PHAGE_Helico_KHP30_NC_019928: DNA helicase; PP_00965; phage(gi431810547)	2.00E-38
8	904588..905757	PHAGE_Helico_KHP30_NC_019928: putative DNA helicase, putative DNA repair protein;PP_00966; phage(gi431810548)	
9	905768..907327	PHAGE_Helico_KHP30_NC_019928: putative primase; PP_00967; phage(gi431810549)	0
10	907324..908997	PHAGE_Helico_KHP30_NC_019928: hypothetical protein; PP_00968; phage(gi431810550)	0
11	909058..914190	PHAGE_Helico_KHP30_NC_019928: hypothetical protein; PP_00969; phage(gi431810551)	0
12	914563..915150	PHAGE_Helico_KHP30_NC_019928: hypothetical protein; PP_00970; phage(gi431810552)	1.00E-97
13	915385..915945	PHAGE_Helico_KHP30_NC_019928: hypothetical protein; PP_00971; phage(gi431810553)	5.00E-98
14	915956..917101	PHAGE_Helico_KHP30_NC_019928: structural protein; PP_00972; phage(gi431810554)	0
15	917115..917477	PHAGE_Helico_KHP30_NC_019928: hypothetical protein; PP_00973; phage(gi431810555)	9.00E-43
16	917528..917959	PHAGE_Helico_KHP30_NC_019928: hypothetical protein; PP_00974; phage(gi431810556)	6.00E-70
17	918030..919418	PHAGE_Helico_KHP30_NC_019928: putative portal protein; PP_00975; phage(gi431810557)	0
18	complement(919375..920658)	PHAGE_Clostr_c_st_NC_007581: putative IS transposase (OrfB); PP_00976; phage(gi80159731)	9.00E-57
19	920727..921155	PHAGE_Clostr_c_st_NC_007581: putative transposase; PP_00977; phage(gi80159828)	3.00E-18
20	921347..921733	PHAGE_Helico_KHP30_NC_019928: putative portal protein; PP_00978; phage(gi431810557)	3.00E-60
21	921694..921840	hypothetical; PP_00979	0
22	921833..922228	PHAGE_Helico_KHP30_NC_019928: putative terminase; PP_00980; phage(gi431810558)	2.00E-67
23	922234..923388	PHAGE_Helico_KHP30_NC_019928: putative terminase; PP_00981; phage(gi431810558)	0
24	923443..923652	PHAGE_Helico_KHP30_NC_019928: hypothetical protein; PP_00982; phage(gi431810559)	4.00E-27
25	923664..923879	PHAGE_Helico_KHP30_NC_019928: hypothetical protein; PP_00983; phage(gi431810560)	4.00E-08
26	923879..924205	PHAGE_Helico_KHP30_NC_019928: putative holin; PP_00984; phage(gi431810561)	7.00E-42
27	924272..924592	PHAGE_Helico_KHP30_NC_019928: hypothetical protein; PP_00985; phage(gi431810563)	4.00E-33
28	924640..925185	PHAGE_Helico_KHP30_NC_019928: hypothetical protein; PP_00986; phage(gi431810564)	2.00E-97
29	925187..925984	PHAGE_Helico_KHP30_NC_019928: hypothetical protein; PP_00987; phage(gi431810565)	4.00E-134
30	925984..926556	PHAGE_Helico_KHP30_NC_019928: hypothetical protein; PP_00988; phage(gi431810566)	3.00E-88
31	926595..926882	PHAGE_Helico_KHP30_NC_019928: hypothetical protein; PP_00989; phage(gi431810567)	6.00E-39
32	926935..927267	PHAGE_Helico_KHP30_NC_019928: hypothetical protein; PP_00990; phage(gi431810568)	2.00E-12
33	927288..928121	PHAGE_Helico_KHP30_NC_019928: hypothetical protein; PP_00991; phage(gi431810569)	7.00E-125
34	928121..928903	PHAGE_Helico_KHP30_NC_019928: hypothetical protein; PP_00992; phage(gi431810570)	4.00E-133
35	933718..933729	attR TCAAAAAACCAC	0

### Virulence genes in Yunnan isolates

**Fig 6:**
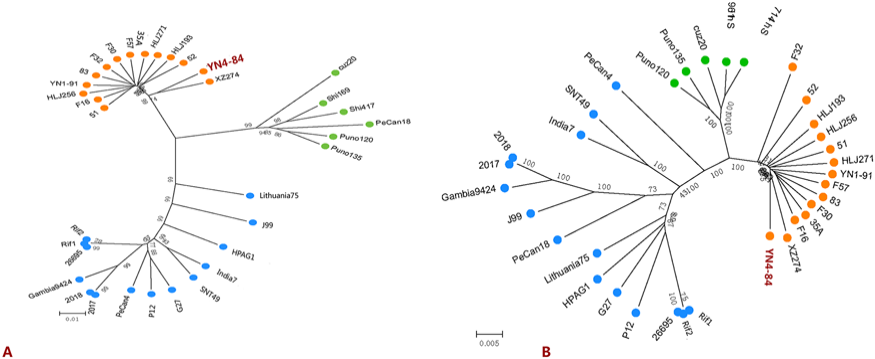
Neighbor-joining tree based on the sequence diversity of *cagA* (Left) and *cagPAI* (Right). YN4–84 is highlighted in red.

**Fig 7:**
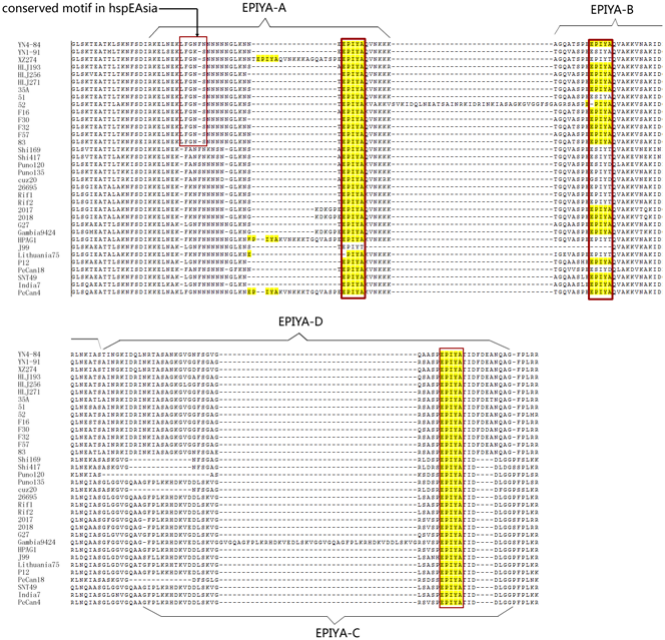
EPIYA motif analysis of the *CagA* C-terminal region.

*CagPAI* from YN4–84 is 37,296 bp in length and has 26 cag protein encoding genes. The *cagA* gene is 3,549 bp in length. *CagPAI* from YN1–91 is 37,492 bp in length and has 26 *cag* protein encoding genes. The *cagA* gene is 3,525 bp in length. For the 47 genome sequences, only 34 had intact *cagPAI* genes with an identical sequence length. From the NJ tree constructed based on *cagA* and *cagPAI*, quite a similar phylogenetic relationship was observed, except for minor differences in the bootstrap value of some branches (Fig. 6). YN4–84 was related to another strain isolated from Tibet in China, which was consistent with the results of the phylogenetic analysis based on the core genome SNPs. YN1–91 was related to other hspEAsia strains. Analysis of the EPIYA motif showed that both YN4–84 and YN1–91 belonged to the EPIYA-A/B/D type. Unexpectedly, we found a conserved motif LFGNS in the left flanking regions of EPIYA-A in all of the analyzed hspEAsia subgroup strains (Fig. 7). Other previously reported possible virulence genes like *sab*A (887355–889637), *bab*A (1528133–1526690), *vac*A (1179426–1183640) and *ice*A1 (1386799–1384980) were also found in YN4–84, while *dup*A and iceA2 were absent.

## Discussion

Genomic features of *H*. *pylori* strains recovered from various geographic regions and ethnic groups is a topic of considerable interest. Even though dozens of strains have complete genome sequences, few reports of isolates recovered from high altitude areas exist. Our previous analysis indicated that Yunnan isolates have significantly different genetic features compared to isolates from other areas, especially for the Naxi population, one of the largest ethnic minorities in Yunnan. To enhance our knowledge of the genomic features of the Naxi isolates, we initiated this genome sequencing project.

One of our most striking findings is a putative intact phage sequence in the Naxi isolate YN4–84. *Helicobacter pylori* was considered devoid of prophages until 2012 when the presence of an incomplete prophage sequence in strain B38 and a complete prophage sequence in strain B45 were reported. Since then, several reports described prophage sequences found in *H*. *pylori* isolates. For example, the phage KHP30 and KHP40 were isolated from culture supernatants of East Asian-type isolates from Japanese patients living in distinct geographic regions and the temperate bacteriophage 1961P was found in a lysate of a clinical strain of *H*. *pylori* isolated in Taiwan [39–42]. In this study, we report an intact phage sequence found in a Chinese *H*. *pylori* isolate for the first time. YN4–84P was inserted between two putative virulence genes, *oip*A and *hom*B (Fig. 4). The HomB protein was expressed in the *H*. *pylori* outer membrane and was antigenic in humans. *H*. *pylori hom*B knockout mutant strains had a reduced ability to induce interleukin-8 secretion in human gastric epithelial cells, as well as a reduced capacity to bind to the cells, which suggests that HomB is involved in the inflammatory response and in *H*. *pylori* adherence [43–45]. OipA, another member of the *H*. *pylori* outer membrane protein family similar to HomB, has been called the “outer inflammatory protein” because of its association with increased interleukin (IL)–8 secretion from epithelial cells *in vitro* and heightened gastric inflammation *in vivo* [46,47]. Both HomB and OipA are surface-exposed adherence factors that can mediate the interactions of *H*. *pylori* with the host microenvironment. The presence of a prophage inserted into this region suggests that HomB and OipA may act as a receptor for YN4–84P. According to the results from PHAST, YN4–84 has a high sequence homology with the other three previously reported *H*. *pylori* phages KHP30, KHP40 and 1961P. These phages were all isolated from *H*. *pylori* isolates of the hspEAsia subgroup. This phenomenon preliminarily indicates that the host bacteria infected by phage may be population specific for *H*. *pylori* species and that the subgroup of *H*. *pylori* hspEAsia may only be sensitive to specific phages like YN4–84P or KHP30, etc. We also found a transformable fragment (IS605) inserted into a putative phage portal protein sequence (Fig. 5), which is rare and different from a previous report that described an IS605 fragment inserted between a hypothetical protein HPF16_0942 and a putative site-specific integrase-resolvase HPF16_0945 in an incomplete, questionable phage in F16 [41]. The distribution of IS605 in *H*. *pylori* phages should be further investigated and characterized. This study reported a phage in a Chinese *H*. *pylori* isolate and its complete genome sequence, however there are still several interesting questions to clarify. Can the phage be released during bacterial growth, or can it be induced by other physical or chemical methods? What is the role of phage in the microevolution or pathogenesis of the *H*. *pylori* hspEAsia subgroup? We are currently undertaking a series of further comprehensive experiments to clarify these issues.

We primarily used four sets of SNPs data for the phylogenetic analyses in this study, including clustering based on the sequence diversity of seven commonly used house-keeping genes, the core genome, the *cagA* gene and the *cagPAI*. All the results showed a similar phylogenetic profile for the tested strains; however, core genome SNP analysis had higher bootstrap values for some branches compared to cluster results based on house-keeping genes, which illustrated that phylogenetic analysis based on global genomic diversity might be more reliable than local alignment. There was also a minor difference for the genetic relationship of YN4–84. Fig. 1B (left) shows that YN4–84 is related to F32 (a Japanese isolate), and YN1–91 is related to 51(a Korean isolate). While in Fig. 1B (right), YN4–84 is more closely related to XZ274 (a Chinese Tibetan isolate) and YN1–91 is more closely related to HLJ271(a Han isolate). It is noteworthy that geographically, Yunnan neighbors Tibet. We are more confident in the results based on core genome SNPs, which were confirmed by the relationships among the other three Chinese Heilongjiang isolates. In the core genome SNPs tree, they are related to 51 (a Korean isolate). Heilongjiang is located in the northeast of China and neighbors Korea. Therefore, it seems that these isolates can be grouped more precisely according to core genome SNPs though they all belong to hspEAsia subgroup. Both YN1–91 and HLJ271 are Han isolates, from the core genome SNPs phylogenetic tree, YN1–91 is close to HLJ271. While from the seven house-keeping genes SNPs tree, YN1–91 is close 51. The results further emphasize that it is more accurate to construct phylogenetic relationship using core genome SNPs.

The core gene number found in this study was 1059, which is a bit lower than that in previous reports [25, 26]. It is unquestionable that, given the increasing number of sequenced *H*. *pylori* isolates based on next-generation sequencing technology, the number of core genes will slightly but gradually decrease. However, according to several estimates from previous studies, the extreme core gene contents cannot be less than 1000 if the genome structure stability for bacterial survival is to be maintained [27–29].

From the global overview of genomic diversity among YN4–84, YN1–91 and the other 45 *H*. *pylori* genome sequences, three genomic regions were found to have dramatic genetic divergence in YN4–84. These regions mainly encode type four secretion system three (TFS3), type four secretion system four (TFS4), and a serine threonine kinase protein. YN4–84 has an intact TFS3 and partial TFS4 system, while YN1–91 has incomplete TFS3 and TFS4 systems. The three Heilongjiang isolates all lack these two systems. Although the core genome SNPs tree reflects the close phylogenetic relationship of YN1–91 and HLJ271 for they all belong to Han population, the climate, geographic environment and habitant lifestyle are quite different for these areas. Whether the absence of genomic TFS3 and TFS4 systems are due to the geographic and environmental distinctions needs to be further explored from the genomic features of more isolates from these area. Some other relatively short variable regions that were not labeled along the chromosome circle mainly included genes encoding type I, type II and type III restriction modification systems. These results are consistent with previous genomic studies and further emphasize the contribution of these ‘plasticity zones’ to external niche adaption and pathogenesis for *H*. *pylori* [32,48,49]. However, there are still few reports that indicate correlation of these regions with geographic differences or disease clinical outcomes except for *cagPAI*. Further studies should focus on the functional analysis of these gene clusters to explore potential mechanisms.

From the phylogenetic tree based on *cagA* and *cagPAI* sequence diversity, 34 strains were divided into three groups correlating with hspEAsia (orange dots), hspAmerind (green dots) and hpEurope (blue dots) (Fig. 6). YN4–84 was shown to be related to XZ274, which was consistent with the results from the core genome SNPs and suggested that the genetic diversity of *cagA* could be used to construct a phylogenetic tree to define strain relationships instead of *cagPAI*, house-keeping genes or core genomes in *cagA* positive isolates. Further comparison of the EPIYA motifs of *cagA* shows that YN4–84 has a EPIYA-A/B/D profile, which is a characteristic of eastern isolates. We also found a conserved LFGNS motif in the left flanking regions of EPIYA-A present in all analyzed hspEAsia strains (Fig. 7). Therefore, we think that this motif is a potential marker to identify *H*. *pylori* isolates that belong to the hspEAsia subgroup, though more strains should be screened to confirm this. We also briefly investigated other important virulence genes in YN4–84, such as *sab*A, *bab*A, *vac*A and *ice*A1, which were present in YN4–84. Sequence analysis of *vac*A revealed that it belonged to the *s1m2* gene type, which is a predominant vacA gene type in the hspEAsia subgroup and is considered characteristic of highly-virulent strains [50, 51]. YN4–84 did not contain *dup*A and *iceA2*. To further investigate the correlation between virulence gene diversity and disease status, more Yunnan isolates are necessary.

In summary, the genome sequencing of Naxi isolate YN4–84 and Han isolate YN1–91 provided useful information for a deep exploration of genetic variations among different *H*. *pylori* populations. High genomic diversity and presence of a phage sequence were found in YN4–84 compared to YN1–91 and genomes from strains found worldwide. In future studies, additional Naxi isolates should be sequenced to explore the potential microevolution mechanism for this unique population.
